# Supplementary material for: Germline cis variant determines epigenetic regulation of the anti-cancer drug metabolism gene dihydropyrimidine dehydrogenase (DPYD)
Source: bioRxiv. 2023 Nov 4:2023.11.01.565230. Preprint. [Version 1] doi: 10.1101/2023.11.01.565230 (PMC10635067; doi:10.1101/2023.11.01.565230)
Supplement: Supplementary file 1 [file NIHPP2023.11.01.565230v1-supplement-1.pdf]

bioRxiv preprint doi: https://doi.org/10.1101/2023.11.01.565230; this version posted November 5, 2023. The copyright holder for this preprint (which was not certified by peer review) is the author/funder, who has granted bioRxiv a license to display the preprint in perpetuity. It is made available under aCC-BY 4.0 International license.

SUPPLEMENTARY TABLES

Supplementary Table S1: Primers used for ChIP, cloning, qPCR, and site-directed mutagenesis.

Target	Forward Primer (5’-3’)	Reverse Primer (5’-3’)
E9 ChIP-1	CCTACCTACCACCCCCAAGA	TGCCCAGGACATTACACATGA
E9 ChIP-2	CATCAGGTTGCTTTTTGCAGC	TTAGCGTGGACTACCAGGGA
E9 ChIP-3	TGGGACTCAAAAAGCGGTTCA	GGTGTTGCGGGTGCTGATTA
E9 ChIP-4	GGTTCAACAGAGGATGCAACAC	AGGGAAAGATTTTCCTGGCCC
E9 ChIP-5	TAAAGATGCACCGAGGTGGG	AGTGTCTGGATTTAAGTAGATGTGC
E9 ChIP-6	AGCAGGAATGAGAAGGAGAGAAG	TCAGTCTCTCACTCCAAACCC
E9 ChIP-7	GCTCTTTTCATTGAAGCCTAAAACA	CCATCATACTTTTTCCAATTGTTGC
E9 ChIP-8	AATGATGAGAGGAAGATGACAAAGT	TGCCTACGCGATGAGTTGTA
DPYD promoter	AGTCGATATCCACAGTGTCTGTGTCTGGC	AGTCAAGCTTGCTCGATGTCCGCCGAG
E9 region	AGTCGGTACCGAATAAAACCAAAATAAAATCCATTTGGACGTTT	AGTCGAGCTCTTTGTGCAAAGGACCTTGGTATTTCC
Rs4294451 A>T	AAAGAAAAATAAATAAAAAAAGGAAAAATCTATAAGC	TTCTGGGGGTTGGTGTTG
Cas9	AACAGCCGCGAGAGAATGAA	CACGGGGTGTTCTTTCAGGA
DPYD	GTAAGGACTCGGCGGACATC	GCCGAAGTGGAACACAGAGT
L32	CCTTGTGAAGCCCAAGATCG	TGCCGGATGAACTTCTTGGT
CEBPB	CGCCGCCTGCCTTTAAATC	AAGCAGTCCGCCTCGTAGTA

bioRxiv preprint doi: https://doi.org/10.1101/2023.11.01.565230; this version posted November 5, 2023. The copyright holder for this preprint (which was not certified by peer review) is the author/funder, who has granted bioRxiv a license to display the preprint in perpetuity. It is made available under aCC-BY 4.0 International license.

Supplementary Table S2. Sequences and positions of the primers used for 3C analysis.

**Primer position (TSS: +1)**	**Sequences (5'-3')**
-22822	GGGAAGTTGAGAGAGCTAGGC
-19322	TGCTCTGTCAGCTGAGAAGACCTAGA
-16420	GTCACTACTGGGACTCTGAGAAA
-15616	AAAAGAAATTGCAACCTCTGGCA
-11097	GTTGCTTTTTGCAGCTGGGAT
-8087 (also used as anchor for E9 region)	AGTGCTTGAAGCTGATGAAGGG
-5884	CTGCAGAACAAGAACAGCACAT
1280 (also used as anchor for promoter)	TTAGGGTAGTCTATTCCTTTTTGGT
2826	TGCTTTGTGAGTGTACTGTTTGG
3076	CCTCCACCGGCAAGGATAAT

bioRxiv preprint doi: https://doi.org/10.1101/2023.11.01.565230; this version posted November 5, 2023. The copyright holder for this preprint (which was not certified by peer review) is the author/funder, who has granted bioRxiv a license to display the preprint in perpetuity. It is made available under aCC-BY 4.0 International license.

SUPPLEMENTARY FIGURES AND FIGURE LEGENDS

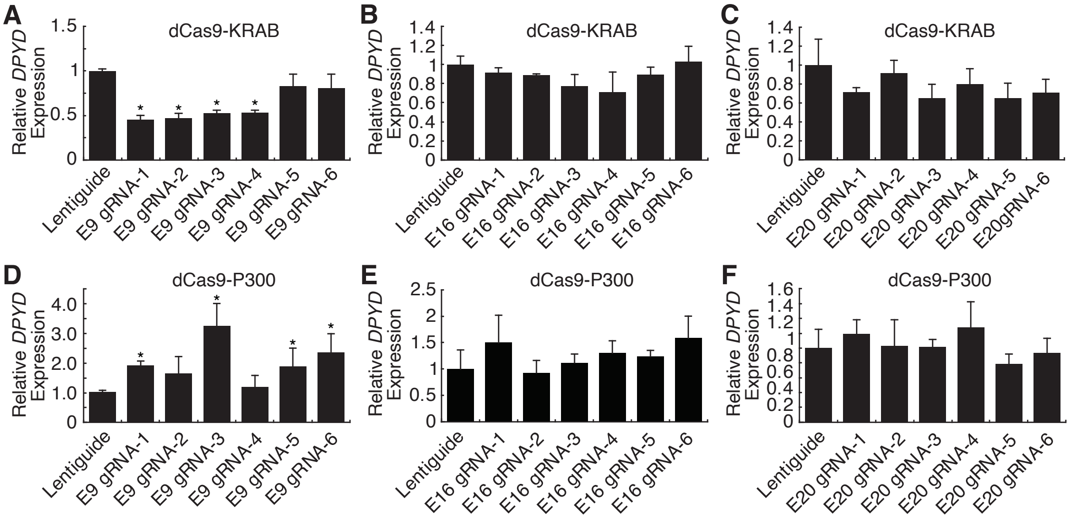

Zhang et al., Figure S1

Figure S1. CRISPRi and CRISPRa screen to identify *DPYD cis* regulatory elements in HCT116 cells. For CRISPRi, *DPYD* expression was measured in HCT116 cells expressing dCas9-KRAB following transfection with guide-RNAs specific to the E9 (A), E16 (B), and E20 (C) regions. For CRISPRa, *DPYD* expression was measured in HCT116 cells expressing dCas9-P300 following transfection with guide-RNAs specific to E9 (D), E16 (E), and E20 (F). Data represent the mean of three independent biological replicates ± SD. *, $p<0.05$. P-values were calculated using two-tailed Student's t-test comparing results to those from lentiguide controls.

bioRxiv preprint doi: https://doi.org/10.1101/2023.11.01.565230; this version posted November 5, 2023. The copyright holder for this preprint (which was not certified by peer review) is the author/funder, who has granted bioRxiv a license to display the preprint in perpetuity. It is made available under aCC-BY 4.0 International license.

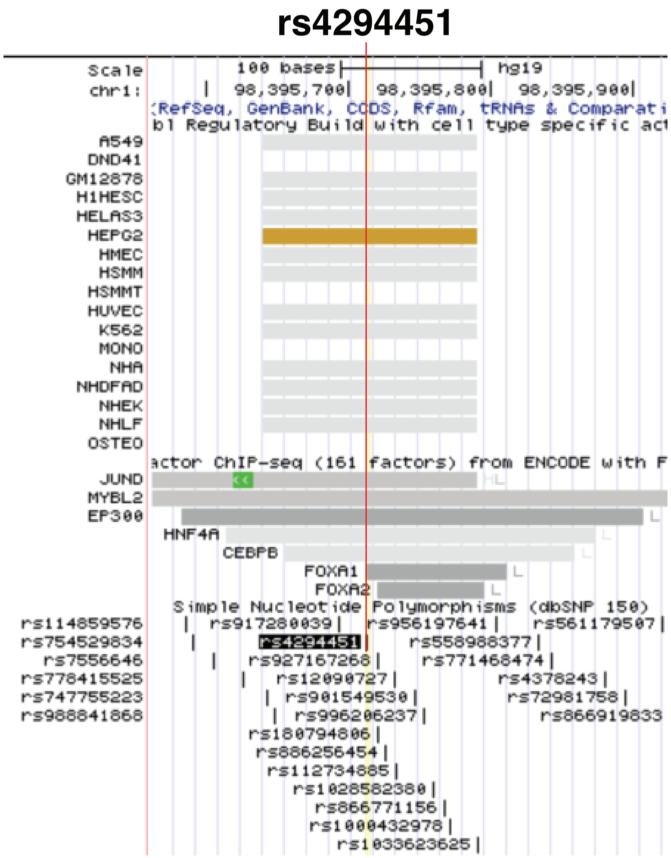

Zhang et al., Figure S2

Figure S2. Genomic context of rs4294451. Rs4294451 is located within a putative enhancer region showing evidence for regulatory activity in Ensembl Regulatory Build data and within transcription factor binding sites in ENCODE Factorbook data.

bioRxiv preprint doi: https://doi.org/10.1101/2023.11.01.565230; this version posted November 5, 2023. The copyright holder for this preprint (which was not certified by peer review) is the author/funder, who has granted bioRxiv a license to display the preprint in perpetuity. It is made available under aCC-BY 4.0 International license.

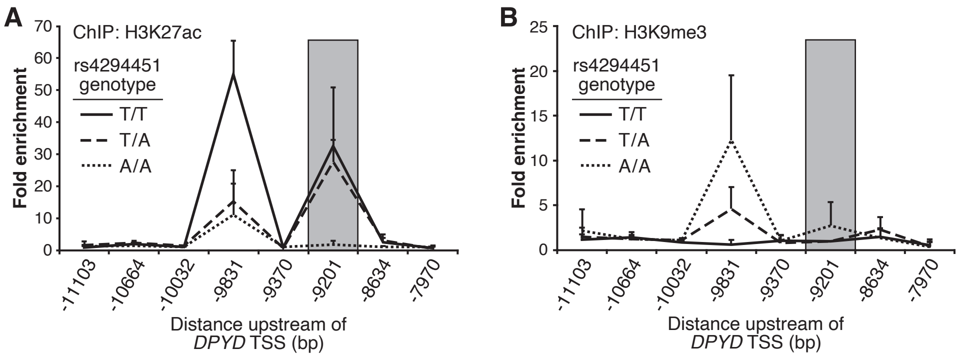

Zhang et al., Figure S3

Figure S3. Rs4294451 A allele is associated with epigenetic repression at the E9 region in human liver specimens. Chromatin enrichment of H3K27ac (A) and H3K9me3 (B) was measured using ChIP-qPCR of liver specimens obtained from human donors carrying different rs4294451 genotypes. Data represent three independent measurements from a single liver specimen with each indicated genotype ± SD.

bioRxiv preprint doi: https://doi.org/10.1101/2023.11.01.565230; this version posted November 5, 2023. The copyright holder for this preprint (which was not certified by peer review) is the author/funder, who has granted bioRxiv a license to display the preprint in perpetuity. It is made available under aCC-BY 4.0 International license.

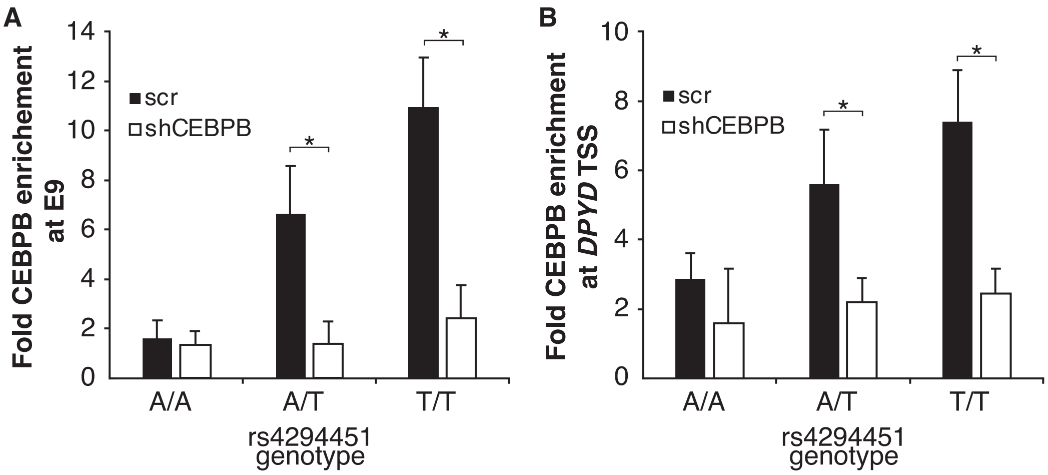

Zhang et al., Figure S4

Figure S4. Disruption of CEBPB reduces enrichment at the E9 region and *DPYD* promoter in rs4294451 T/T and A/T cells, but not in T/T cells. CEBPB enrichment at the E9 region (A) and the *DPYD* promoter (B) was measured by ChIP-qPCR in CEBPB knockdown and scramble (scr) control knock-in HCT116 cells containing the indicated rs4294451 genotype. Data represent the mean of three independent replicates ± SD. *, $p<0.05$. P-values were calculated using two-tailed Student's t-test.

bioRxiv preprint doi: https://doi.org/10.1101/2023.11.01.565230; this version posted November 5, 2023. The copyright holder for this preprint (which was not certified by peer review) is the author/funder, who has granted bioRxiv a license to display the preprint in perpetuity. It is made available under aCC-BY 4.0 International license.

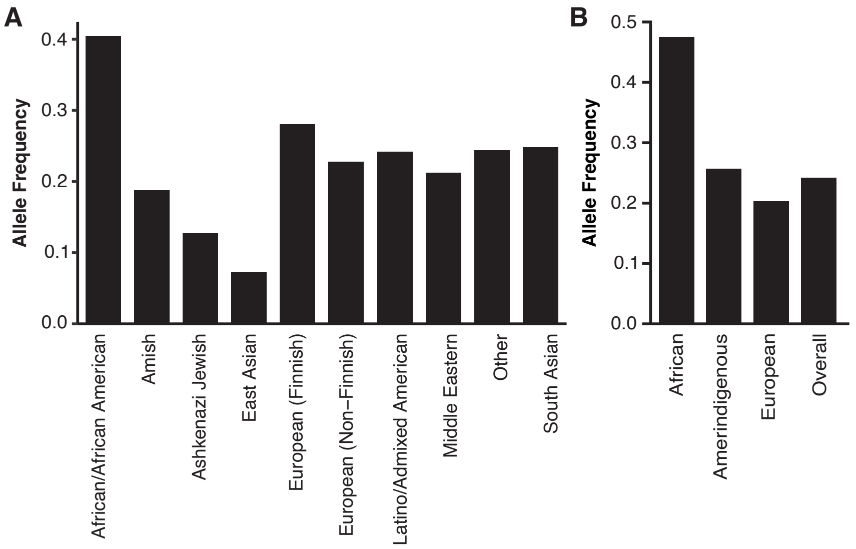

Figure S5. Allele frequency for rs4294451-T allele in global populations. (A) Allele frequencies in various populations for the rs4294451-T allele was retrieved from the gnomAD browser v3.1.2. (B) Local ancestry-informed frequency data was retrieved for the rs4294451-T allele within Latino-Admixed American samples of gnomAD v3.1
